# Improved synthesis of SV2A targeting radiotracer [^11^C]UCB-J

**DOI:** 10.1186/s41181-019-0080-5

**Published:** 2019-11-29

**Authors:** Johanna Rokka, Eva Schlein, Jonas Eriksson

**Affiliations:** 1 Department of Public Health and Caring Sciences/Geriatrics, Uppsala University, Rudbeck Laboratory, 751 85 Uppsala, Sweden; 2 Department of Medicinal Chemistry, Uppsala Biomedical Center, Uppsala University, SE-751 23 Uppsala, Sweden; 3 PET Centre, Uppsala University Hospital, SE-751 85 Uppsala, Sweden

**Keywords:** [^11^C]UCB-J, SVA2, Synaptic density, PET, Synaptic vesicle glycoprotein, Suzuki-Miyaura coupling, ^11^C-methylation

## Abstract

**Introduction:**

[^11^C]UCB-J is a tracer developed for PET (positron emission tomography) that has high affinity towards synaptic vesicle glycoprotein 2A (SV2A), a protein believed to participate in the regulation of neurotransmitter release in neurons and endocrine cells. The localisation of SV2A in the synaptic terminals makes it a viable target for in vivo imaging of synaptic density in the brain. Several SV2A targeting compounds have been evaluated as PET tracers, including [^11^C]UCB-J, with the aim to facilitate studies of synaptic density in neurological diseases.

The original two-step synthesis method failed in our hands to produce sufficient amounts of [^11^C]UCB-J, but served as an excellent starting point for further optimizations towards a high yielding and simplified one-step method. [^11^C]Methyl iodide was trapped in a clear THF-water solution containing the trifluoroborate substituted precursor, potassium carbonate and palladium complex. The resulting reaction mixture was heated at 70 °C for 4 min to produce [^11^C]UCB-J.

**Results:**

After semi-preparative HPLC purification and reformulation in 10% ethanol/phosphate buffered saline, the product was obtained in 39 ± 5% radiochemical yield based on [^11^C]methyl iodide, corresponding to 1.8 ± 0.5 GBq at EOS. The radiochemical purity was > 99% and the molar activity was 390 ± 180 GBq/μmol at EOS. The product solution contained < 2 ppb palladium.

**Conclusions:**

A robust and high yielding production method has been developed for [^11^C]UCB-J, suitable for both preclinical and clinical PET applications.

## Introduction

The synaptic vesicle glycoprotein 2A (SV2A) is localized at synaptic vesicles in neurons and endocrine cells where it is believed to participate in the regulation of neurotransmitter release. The importance of SV2A for synaptic function is indicated for example by the antiepileptic action of levetiracetam (Lynch et al. 2004) which targets the protein, and by the development of seizures in knockout mice lacking SV2A (Janz et al. 1999). The localisation and apparent function of SV2A makes it a promising proxy marker for synaptic density that could be used to study neurodegenerative and psychiatric diseases in the brain (Finnema et al. 2016; Chen et al. 2018; Koole et al. 2019).

Several compounds with high affinity for SV2A, labelled with carbon-11 or fluorine-18, have been investigated as tracer candidates for in vivo quantification of SV2A with positron emission tomography (PET) (Heurling et al. 2019). One of the more promising candidates, [^11^C]UCB-J, was discovered by UCB Pharma (Braine-l’Alleud, Belgium) among 500+ compounds screened for binding affinity and was identified to have properties suitable for SV2A PET (Mercier et al. 2014). Preclinical evaluation at Yale PET centre revealed that [^11^C]UCB-J was indeed a promising ligand for SV2A imaging (Nabulsi et al. 2016) and recent publications have shown that [^11^C]UCB-J is a suitable PET tracer also for human studies (Finnema et al. 2016; Chen et al. 2018; Koole et al. 2019; Holmes et al. 2019). The original [^11^C]UCB-J synthesis was performed as a two-step reaction by ^11^C-methylation with [^11^C]methyl iodide under Suzuki-Miyaura coupling conditions (Nabulsi et al. 2016). [^11^C]Methyl iodide was reacted with a palladium (0) catalyst followed by addition and hydrolysis in situ of a trifluoroborate precursor to activate it towards palladium mediated ^11^C-methylation.

The Suzuki-Miyaura coupling can be described in three mechanistical steps; *oxidative addition* of an organohalide or triflate lacking beta-hydrogens to palladium (0), *transmetalation* of organoborane followed by a *reductive elimination* step where the product is formed. A suitable catalyst is the [(o-Tol)_3_P-Pd-P(o-Tol)_3_] complex, with a large cone angle (194°) appropriate for transmetalation (Schubiger et al. 2007). However, palladium mediated ^11^C-methylation differs from the general scheme of transition-metal-catalysis in the sense that the reaction is foremost optimized to consume the ^11^C-labelled precursor, in this case [^11^C]methyl iodide, while all other reagents and catalyst materials are employed in large excess, de facto ruling out a propagating catalytic cycle. The prerequisite of a short reaction time, necessitated by the rapid physical decay of carbon-11 (T_1/2_ = 20.4 min) is further helped by the very low quantities of [^11^C]methyl iodide used in the reaction (nano mole scale). A single clinical PET investigation in human typically requires a few hundred MBq of a ^11^C-tracer ready for injection and significantly more for multiple investigations. When we implemented the original [^11^C]UCB-J synthesis procedure, the method failed to give a sufficiently high radioactivity yield. After unsuccessful attempts to optimize the reaction in DMF-water we turned to another biphasic solvent mixture, THF-water, that efficiently facilitates hydrolysis of trifluoroborates while potentially supressing protodeboronation and other side reactions (Butters et al. 2010).

Here we describe our investigation towards a significantly improved synthesis of [^11^C]UCB-J utilizing the same trifluoroborate substituted precursor that was described in the original synthesis, albeit obtained from a commercial vendor with high chemical purity. The result was a simplified and robust synthesis method performed in a single step which produced [^11^C]UCB-J in 39 ± 5% radiochemical yield (RCY) based on [^11^C]methyl iodide.

## Methods

Reaction mixtures were degassed with helium and vortexed before use. All reported radiochemical yields are decay corrected and based on [^11^C]methyl iodide.

### Materials

The precursor for [^11^C]UCB-J synthesis, (*R*)-3-(difluoroboranyl)-4-((2-oxo-4-(3,4,5-trifluorophenyl) pyrrolidin-1-yl) methyl)-pyridin-1-ium fluoride (BF_3_-Dm-UCB-J) and the reference compound (4*R*)-1-[(3-methyl-4-pyridyl) methyl]-4-(3,4,5-trifluorophenyl) pyrrolidin-2-one (UCB-J), were purchased from Pharmasynth (Tartu, Estonia). Lithium aluminum hydride in THF (0.1 M, LAH) was purchased from ABX (Radeberg, Germany). Tris(dibenzylideneacetone)-dipalladium (0) (Pd_2_(dba)_3_), tri(o-tolyl)-phosphine (P(o-tol)_3_), anhydrous potassium carbonate, *N, N*-dimethylformamide (DMF), acetonitrile (ACN), 37% ammonia solution, phosphorous pentoxide (Sicapent), Ascarite, hydriodic acid (57%), trifluoroacetic acid (TFA) and tetrahydrofuran (THF) were purchased from Sigma Aldrich (Stockholm, Sweden). THF was distilled from a mixture containing sodium and benzophenone just before use to remove water and peroxides. Aqueous solution of TFA (1%) was prepared by dilution with MilliQ water. Ethanol (99.5%) was from Kemetyl (Haninge, Sweden). Phosphate buffer in saline (PBS, pH 7.4) and Kleptose© (Hydroxypropyl betacyclodextrins 300 mg in 9 mg/mL NaCl solution) were from APL (Stockholm, Sweden). Ethanol-PBS solution (10%) was prepared in house. Ammonium formate (AMF) buffer solution (50 mM, pH 3.5) was purchased from Bio-Hospital (Kopparberg, Sweden). AMF buffer solution (pH 10) was prepared by mixing ammonia solution (40 mL, 37%) and AMF solution (2000 mL, 50 mM, pH 3.5). The ^11^C-methylation reactions were performed in disposable conical glass vials (crimp neck, 0.9 mL) with septa (11 mm aluminium crimp cap with 1.3 mm butyl/PTFE seal), purchased from VWR (Karlskoga, Sweden). Sep-Pak tC18 Plus Light Cartridges (WAT036805) were from Waters. Sterile filter (0.22 μm pore-size, Millex GV) was purchased from Millipore (Solna, Sweden).

## General methods

### Synthesis equipment

The synthesis, purification and reformulation procedures were fully automated using Tracer Production System (TPS) developed in-house (Uppsala University Hospital PET Centre, Sweden). The TPS device contains 10 modules, including a robotic liquid handler, a gripper with a shaker function, heating/cooling for reaction vials, injection port for semi-preparative HPLC, fraction collector, vortex evaporator, SPE reformulation system, sterile dispenser and a control software that allows independent tasks to run concurrently.

### Production of [^11^C]methyl iodide

Carbon-11 obtained as [^11^C]CO_2_ was produced through the ^14^N(p, α)^11^C nuclear reaction by 17 MeV proton irradiation (Scanditronix MC-17 cyclotron) on nitrogen gas (AGA, Nitrogen 6.0) containing 0.05% oxygen (AGA, Oxygen 4.6). Typical irradiation time was 30 min, which gave approximately 30 GBq [^11^C]CO_2_ at the end of bombardment (EOB). The [^11^C]CO_2_ was converted to [^11^C]methyl iodide by the “wet method”. First, [^11^C]CO_2_ was transferred to a reactor using helium gas (100 mL/min) and trapped in LAH (300 μL, 0.1 M). The mixture was evaporated to dryness at 80 °C under a stream of nitrogen gas (150 mL/min) during 2 min. Hydroiodic acid (600 μL, 57%) was added to the reactor and mixture was heated at 130 °C for 70 s and then cooled to 80 °C. The formed [^11^C]methyl iodide was then transferred in a stream of nitrogen gas (10 mL/min) to the reaction vessel via a phosphorous pentoxide/Ascarite column to remove moist and acidic vapors. Using this method, 18.3 ± 3.6 GBq [^11^C]methyl iodide was produced (*n* = 20).

### Product purification

Purification of [^11^C]UCB-J was performed by semi preparative HPLC (Agilent 1260 Infinity II) using a Phenomenex Gemini 5 μm NX-C18 110 Å (250 × 10 mm) column eluted with 40% ACN in AMF buffer (pH 10) at 5 mL/min. The effluent was monitored with a Bioscan Flow-Count PMT radioactivity detector and UV detector (Agilent 1260 Infinity II) set at 254 nm. The fraction containing [^11^C]UCB-J was collected at a retention time of approximately 10–11 min.

### Radiochemical and chemical purity, identity and molar activity

The radiochemical purity (RCP) and concentration of formulated [^11^C]UCB-J was assessed by analytical HPLC (VWR LaChrom Elite) equipped with an auto-sampler (L-2200) and pump (L-2130). A Phenomenex Kinetex 5 μm C18 100 Å (100 × 3.0 mm) column was eluted with 30% ACN in AMF buffer (pH 3.5) at 0.7 mL/min. The effluent was monitored with a radioactivity detector (Bioscan Flow-Count PMT) and UV diode array detector set at 229–279 nm. The identity of [^11^C]UCB-J was assessed by comparing the retention time of radioactive peak with the retention time of the UV peak for UCB-J, a non-radioactive reference compound added to the product sample. The molar activity was determined from the concentration of [^11^C]UCB-J and the radioactivity was measured in a dose calibrator and decay-corrected to end of synthesis (EOS). No chemical impurities were observed by UV-HPLC. The palladium concentration was analyzed in three batches of formulated product produced by the one-step synthesis. The palladium analyses were performed at the Department of Chemistry – BMC at Uppsala University using inductively coupled plasma atomic emission spectroscopy (ICP-AES).

### Synthesis of [^11^C]UCB-J

#### Original method, two-step synthesis in dmf-water (Method I)

The original synthesis method for [^11^C]UCB-J (Nabulsi et al. 2016; Holmes et al. 2019) was implemented with minor modifications using the automated TPS synthesis system. Pd(dba)_3_ (0.4 mg), P(o-tol)_3_ (0.4 mg) and potassium carbonate (0.8 mg) were dissolved in DMF:water (250 μL, 8:1 v/v) forming an inhomogeneous dark red solution that gradually turned yellowish. After addition of [^11^C]methyl iodide in a stream of nitrogen gas, the resulting mixture was heated at 30 °C for 3 min with brief and vigorous shakes at 1 and 2 min. The precursor (1.2 mg, *n* = 3; or 3 mg, *n* = 2) in DMF:water (100 μL, 8:1 v/v) was then added and the resulting solution was heated at 100 °C for 5 min while the vial was shaken briefly every 30 s. The reaction vial was then cooled to 50 °C prior dilution of the reaction mixture with water (600 μL). Purification of [^11^C]UCB-J was performed by semi-preparative HPLC. The collected HPLC fraction containing [^11^C]UCB-J was mixed with Kleptose© (0.3 mL) and trifluoroacetic acid (1 mL). The resulting mixture was transferred to a vortex evaporator where it was evaporated to dryness within 5 min, at a temperature of 20–55 °C. The product was then reformulated by addition of 10% ethanol in PBS (3 mL, pH 7.4). The total synthesis time was 38 ± 3 min (*n* = 5).

#### Two-step synthesis in THF-water (Method II)

Pd_2_(dba)_3_ (0.4 mg) and P(o-tol)_3_ (0.5 mg) were dissolved in THF (250 μL) forming a clear dark red solution that gradually turned yellowish. After addition of [^11^C]methyl iodide in a stream of nitrogen gas at room temperature the resulting solution was heated at 30 °C for 3 min with brief and vigorous shakes at 1 and 2 min. A solution of the precursor BF_3_-Dm-UCB-J (1.1 mg) and K_2_CO_3_ (1.1 mg) in THF (90 μL) and water (40 μL) was then added to the reaction vial. The resulting reaction solution was heated at 70 °C for 5 min and mixed by a vigorous shake of the vial at the start of the reaction and again after 1 min. The [^11^C]UCB-J was purified by semi-preparative HPLC and reformulated by vortex evaporation as described previously. The total synthesis time was 39 ± 6 min (*n* = 7).

#### One-step synthesis in THF-water (Method III)

Pd_2_(dba)_3_ (0.4 mg), P(o-tol)_3_ (0.5 mg), potassium carbonate (1.0 mg) and the precursor BF_3_-Dm-UCB-J (1.1 mg) were dissolved in THF (350 μL) and water (40 μL) and the resulting homogenous dark red solution gradually turned yellowish. Approximately 15 min later, [^11^C]methyl iodide was added to the reaction mixture in a stream of nitrogen gas at room temperature. The resulting solution was heated at 70 °C for 4 min and mixed by a vigorous shake of the vial at the start of the reaction and again after 1 min. The [^11^C]UCB-J was purified by semi-preparative HPLC as described previously. The collected HPLC fraction was mixed with water (20 mL) and passed over a tC18 Sep-Pak cartridge followed by rinsing of the cartridge with water (2 × 20 mL). [^11^C]UCB-J was eluted with ethanol (0.3 mL) followed by PBS (4.5 mL) and sterile filtered into the sterile product vial. The total synthesis time was 39 ± 3 min (*n* = 9).

## Results

**Scheme 1:**
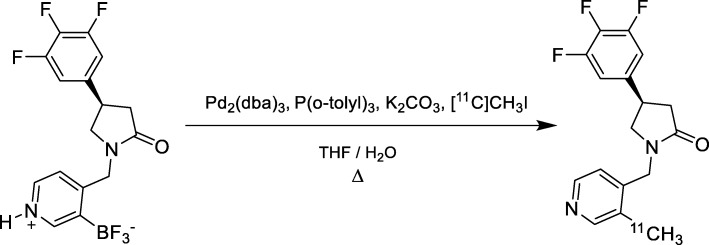
Synthesis of [^11^C]UCB-J (Method III)

The original two-step [^11^C]UCB-J synthesis method gave in our hands less than 1% RCY based on [^11^C]methyl iodide, corresponding to 53 ± 15 MBq (*n* = 3) at EOS, Table 1. Performing the same reaction in THF-water increased the RCY significantly to 16 ± 5% (*n* = 7). In these tests the end-product was formulated using vortex evaporation, where the recovery of the product from the formulation process was only 50%. When performing the synthesis in one-step and formulating the product by solid phase extraction (90% recovery) the RCY was 39 ± 5%, corresponding to 1.8 ± 0.5 GBq at EOS (Scheme 1, Table 1). The molar activity was 390 ± 180 GBq/μmol at EOS and the radiochemical purity was > 99% at 1 h after EOS. The palladium concentration in the product solution was 1.7 ± 0.5 ng/mL (*n* = 3).

**Table 1:** Optimization of the [^11^C]UCB-J synthesis

Method	Reaction steps	[^11^C] CH_3_I[GBq]^c^	Precursor [mg]	Solvent	Reaction temperature	Reaction Time (min)	[^11^C]UCB-J [MBq]^d^	RCY^e^	n^f^
I	2^a^	18 ± 4	1.2 ± 0.1	DMF-H_2_O (8:1)	30 °C + 100 °C	3 + 5	53 ± 15	< 1%	3
I	2^a^	18; 20	3.0; 3.0	DMF-H_2_O (8:1)	30 °C + 100 °C	3 + 5	138; 216	3%; 4%	2
II	2^a^	21 ± 4	1.1 ± 0.1	THF-H_2_O (34:4)	30 °C + 70 °C	3 + 5	890 ± 240	16 ± 5%	7
III	1^b^	17 ± 2	1.1 ± 0.1	THF-H_2_O (35:4)	70 °C	4	1780 ± 460	39 ± 5%	9

^a^Formulated by vortex evaporator method

^b^Formulated by Sep-Pak method

^c^Starting activity

^d^At EOS

^e^RCY based on [^11^C]CH_3_I

^f^Number of experiments

## Discussion

The original synthesis procedure (Nabulsi et al. 2016) failed in our hands to produce sufficient amounts of [^11^C]UCB-J, but served as an excellent starting point for optimizing the reaction conditions. The reaction was performed in two steps where [^11^C]methyl iodide was reacted with a palladium complex formed in a mixture of Pd_2_(dba)_3_), P(o-tol)_3_ and potassium carbonate in DMF-water at ambient temperature. The trifluoroborate substituted precursor was then added and the resulting mixture was heated to produce [^11^C]UCB-J. When modifying the reaction conditions, it was found that a prolonged reaction time did not improve the RCY, nor did an increase of the precursor load from 1 mg to 3 mg or changes in the reaction temperature.

The inhomogeneous character of the prepared reaction mixtures in DMF-water were visually apparent. DMF or DMF-water solutions are commonly used in coupling reactions with [^11^C]methyl iodide and borate precursors in the presence of palladium catalysts (Nabulsi et al. 2016; Holmes et al. 2019; Hamill et al. 2005; Takahashi et al. 2014; Wilson et al. 2018). However, palladium complexes are usually only slightly soluble in DMF-water mixtures and solid particles are easily formed which may result in the precipitation of [^11^C]methylated palladium complexes potentially making them less accessible for reaction.

Mechanistical studies show that trifluoroboronate substrates need to undergo hydrolysis to boronic acid derivatives, [RBF(_3-n_)(OH)_n_]^−^, in order to act as transmetalating species (Butters et al. 2010). As the hydrolysis may occur more efficiently in the minor aqueous phase which is much more basic than the major organic phase, the formation of boronic acid derivatives may also depend on efficient agitation. However, continuous agitation or prefiltration did not improve the RCY of [^11^C]UCB-J in our tests with DMF-water solvent mixtures.

The original synthesis method used a precursor batch which contained approximately 3% of the boronic acid derivative. (Nabulsi et al. 2016; Holmes et al. 2019) Recently another research group published a [^11^C]UCB-J synthesis method where the precursor batch deliberately contained 5% boronic acid derivative (Bertoglio et al. 2019). In addition, a brief note on the synthesis of [^11^C]UCB-J via in situ generation of the boronic acid derivative was published as an abstract for EANM 2017 (Onega et al. 2017). These reports gave further evidence that boronic acids play an important role in the [^11^C]UCB-J synthesis and help to explain why the original synthesis procedure does not perform satisfactorily when applied with high purity batches of the organotrifluoroborate precursor. The UCB-J precursor batch we used was purchased from a commercial manufacturer and had high chemical purity (98.9%). For this reason, we tested to treat the precursor with aqueous carbonate solution to promote hydrolysis before the ^11^C-methylation reaction. However, premixing the precursor with carbonate approximately 30 min before the reaction did not significantly improve the radiochemical yield (3% and 4%, *n* = 2).

Unlike boronic acids, organotrifluoroborates are stable in crystalline solid form and in aprotic solutions making them attractive as precursor materials for synthesis of PET-tracers (Lennox and Lloyd-Jones 2014). However, as already mentioned trifluoroborates need to be converted to boronic acid derivatives in aqueous or protic media to be activated for transmetalation reactions (Lennox and Lloyd-Jones 2014). While conventional catalysis reactions with organotrifluoroborates can proceed for hours, the short half-life of carbon-11 necessitate very short reaction time of a few minutes and rapid initial formation of boronic acid is therefore needed. Boronic acid derivatives have also been used directly as precursors in Suzuki-Miyaura reactions, for example in the synthesis of [^11^C]M-MTEB (Hamill et al. 2005). However, boronic acids can undergo side reactions under coupling conditions, e.g. oxidation, protodeboronation and palladium catalyzed homocoupling. By hydrolyzing trifluoroborates in situ to form moderate concentrations of active species, the reaction can be directed towards the Suzuki-Miyaura coupling reaction more selectively (Lennox and Lloyd-Jones 2014). The hydrolysis rate of trifluoroborates can be controlled in biphasic solvent system that has slightly basic organic phase and very basic aqueous phase. (Lennox and Lloyd-Jones 2014) Besides DMF-water solvent systems, hydrolysis can also occur in more homogenous solvent mixtures such as THF and water in a 10:1 ratio in the presence of a carbonate salt (Lennox and Lloyd-Jones 2014). THF has also shown to be an excellent solvent for other types of transition metal mediated ^11^C-labelling reactions, e.g. carbonylations (Eriksson et al. 2012; Takashima-Hirano et al. 2012; Roslin et al. 2017; Schembri et al. 2019) and Heck reactions (Wang et al. 2015).

When DMF-water was replaced by THF-water in the two step synthesis of [^11^C]UCB-J, the RCY based on [^11^C]methyl iodide was increased from less than 1% to 16 ± 5% (*n* = 7). The optimal reaction temperature was 70 °C, near the boiling point of the solvent and 30 °C lower than the original method (Nabulsi et al. 2016; Holmes et al. 2019). Heating the vial at temperatures over 70 °C did not improve the radiochemical yield but caused higher pressure in the reaction vessel and an increased risk for the vial septum to leak. The effect of the solvent change was visually apparent as THF-water gave clear solutions while DMF-water gave inhomogeneous mixtures containing particulates. Hence, the concentration of the active reagent species in solution may have been enhanced by the higher solubility in THF-water. It could also be speculated that THF-water solvent promoted more rapid initial formation of boronic acid derivatives from the precursor, as well as an improved solvation of the active catalyst complex. Given the lack of preceding studies, further investigations will be needed to confirm the general utility of THF-water as a preferred solvent system for ^11^C-labelling by the Suzuki-Miyaura coupling reaction.

Finally, when the synthesis of [^11^C]UCB-J was performed in a single step, mixing all reagents before adding the [^11^C]methyl iodide, the procedure was greatly simplified and the RCY based on [^11^C]methyl iodide was further improved to 39 ± 5% (*n* = 9), corresponding to 1.8 ± 0.5 GBq at EOS (Table 1). Quality control showed that [^11^C]UCB-J was produced with high radiochemical yield, radiochemical purity and molar activity. The amount of [^11^C]methyl iodide used in the reactions were approximately 20 GBq, with larger starting activity the radioactivity yield of [^11^C]UCB-J could be improved further.

In our investigations, freshly distilled THF over sodium was used because our laboratory has a distillation unit for everyday use. However, when testing the reaction with THF straight from the bottle the RCY remained the same. Thus, it was concluded that freshly distilled THF was not really needed.

In the publications describing the original synthesis of [^11^C]UCB-J, the [^11^C]methyl iodide was trapped in the reaction vessel cooled by an acetone/ice bath (Nabulsi et al. 2016; Holmes et al. 2019). To investigate if cooling was necessary when performing the reaction in THF-water, tests were made showing that more than 80% of [^11^C]methyl iodide was recovered in the reaction vessel at room temperature, hence it was concluded that [^11^C]methyl iodide was trapped efficiently in THF-water without cooling. Another simplification compared to the original method was to omit acidification and filtration and instead rely on separation of palladium species by semi-preparative HPLC. Analysis performed by ICP-AES confirmed that the residual concentration of palladium in the formulated product was insignificant (approx. 2 ppb).

Solid-phase extraction using tC18 light cartridge was preferred over vortex evaporation for the reformulation process using the high yielding one-step synthesis due to excellent product recovery from the formulation process, 90%, based on the activity in the collected HPLC fraction. [^11^C]UCB-J was released from the cartridge using 300 μL ethanol followed by 3 mL PBS solution. High activity concentration was important in an on-going PET study in mice where maximum injection volume was 100 μL and the product solution could contain no more than 10% ethanol.

## Conclusions

[^11^C]UCB-J was synthesized in 39 ± 5% RCY based on [^11^C]methyl iodide, corresponding to 1.8 ± 0.5 GBq at EOS in a one-step reaction from [^11^C]methyl iodide. Besides producing [^11^C]UCB-J in sufficient amounts for clinical PET studies this improved synthesis method present practical simplifications compared to the original method. The ^11^C-methylation reaction was performed in a single step with commercially available precursor at reduced temperature and shortened reaction time. THF-water produced a more homogenous reaction mixture and no filtration was needed prior semi-preparative HPLC. The method has proven robust and several batches of [^11^C]UCB-J has been used in preclinical imaging studies.

## Data Availability

The authors declare that data supporting the findings of this study are available within the article or are available from the corresponding author on reasonable request.
